# Chaperone-mediated autophagy substrate proteins in cancer

**DOI:** 10.18632/oncotarget.17583

**Published:** 2017-05-03

**Authors:** Ying Tang, Xiong-Wen Wang, Zhan-Hua Liu, Yun-Ming Sun, Yu-Xin Tang, Dai-Han Zhou

**Affiliations:** ^1^ Department of Oncology, The First Affiliated Hospital of Guangzhou University of Chinese Medicine, Guangzhou University of Chinese Medicine, Guangzhou 510006, China; ^2^ Department of Gynecology and Obstetrics, Maternal and Child Health Hospital of Zhoushan, Zhoushan 316000, China

**Keywords:** chaperone-mediated autophagy, substrate proteins, cancer, glycolysis, Warburg effect

## Abstract

All intracellular proteins undergo continuous synthesis and degradation. Chaperone-mediated autophagy (CMA) is necessary to maintain cellular homeostasis through turnover of cytosolic proteins (substrate proteins). This degradation involves a series of substrate proteins including both cancer promoters and suppressors. Since activating or inhibiting CMA pathway to treat cancer is still debated, targeting to the CMA substrate proteins provides a novel direction. We summarize the cancer-associated substrate proteins which are degraded by CMA. Consequently, CMA substrate proteins catalyze the glycolysis which contributes to the Warburg effect in cancer cells. The fact that the degradation of substrate proteins based on the CMA can be altered by posttranslational modifications such as phosphorylation or acetylation. In conclusion, targeting to CMA substrate proteins develops into a new anticancer therapeutic approach.

## INTRODUCTION

Autophagy is a tightly regulated catabolic process in which cytoplasmic organelles and proteins are degraded in the lysosome [1]. This process enables cells to retain cellular environmental homeostasis, quality control and energy balance [2, 3]. Studies reveal that autophagy acts as housekeeping functions, can be induced by various stresses to adapt to the conditions of the environment change [4]. There are mainly three different autophagic pathways according to the substrates delivery mode to lysosome, including macroautophagy, microautophagy and chaperone-mediated autophagy (CMA) [5]. CMA and macroautophagy are two well characterized pathways [6, 7]. Macroautophagy is a largely nonselective degradation system, which delivers cytosolic components into a double-membrane structure (autophagosome), and then fuses with lysosome. Whereas CMA is a selective process by which cytosolic substrate proteins bearing a KFERQ-like motif are transported into the lysosome for degradation. This selective degradation is mediated by binding to heat shock 70kDa protein 8 (HSC70) [7], and then substrates become unfolding before delivery into the lysosome by lysosome-associated membrane protein 2A (LAMP2A) [8]. Despite so many differences, the research has found the existence of connection between CMA and macroautophagy, with one compensating for another pathway if it is impaired [9, 10]. In general, microautophagy directly uptakes of cytosolic components into the lysosomal lumen by the invagination [11, 12]. However, the selective degradation system for substrates has also been found in an exceptive form of microautophagy, termed endosomal microautophagy (e-MI). Both CMA and e-MI require a pentapeptide motif related to KFERQ in substrate proteins for binding to HSC70. In contrast with CMA, selective e-MI does not need LAMP2A and proteins unfolding [13]. Consequently, CMA targets and degrades substrate proteins make it quite different from other two autophagic pathways.

In recent years, the researchers are keen on how CMA influences cancer pathophysiology. Kon et al. revealed an increase in the activity of CMA in a variety of cancer cells, with the up-regulated expression of LAMP2A. They also demonstrated that CMA is necessary for malignant cell growth and tumor metastasis [14]. Nevertheless, targeting the CMA in established cancer can inhibit the cancer cells is still being debated. Selective activation of CMA can eliminate cancer cells by inducing the aberrant mutant proteins degradation in the specific cancer cells [15]. Therefore, numerous researchers are particularly interested in the relationship between CMA substrate proteins and cancer biology. Through analysis of the CMA substrate proteins in cancer cells, CMA involves in glucose metabolism [14] and reducing the cellular stress [16]. In this review, we focus on substrate proteins which are degraded by CMA in cancer. We analyze the role of CMA substrate proteins in cancer and CMA substrate proteins are supposed to be developed into a curative approach for anticancer therapy.

## MOLECULAR MECHANISM OF CMA

**Figure 1:**
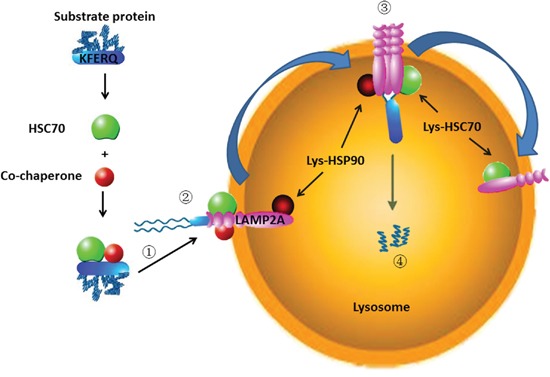
The process of CMA: (1) Recognizing substrate proteins and targeting them to lysosome; (2) Binding and unfolding substrate proteins; (3) Translocation into lysosomes; (4) Degradation by lysosome hydrolytic enzymes

CMA is a kind of selective autophagy, which degrades the cytosolic proteins [17]. The process of CMA can be summarized as follows (Figure 1).

## RECOGNIZING SUBSTRATE PROTEINS AND TARGETING THEM TO LYSOSOME

CMA substrate proteins must contain the amino acid sequence of the polypeptide motif KFERQ [18]. The amino acids consist of a glutamine (Q) residue preceded or followed by the four residues, a basic amino acid of the two positively charged residues, lysine (K) or arginine (R), an acidic amino acid of the two negatively charged residues, glutamic acid (E) or aspartic acid (D), and one or two of these hydrophobic residues, K, R, phenylalanine (F), valine (V), leucine (L) or isoleucine (I) [19]. Nevertheless, in some cases, substrates bearing more than one KFERQ-like motif, it is demonstrated experimentally that additional motif does not change the degradation of the substrates, and one motif is sufficient [20]. The KFERQ-like motif can be buried in both ends or the central domain of the substrate protein. The motif should be exposed or accessible for chaperone recognition. The following conditions can promote the exposure of the KFERQ-like motif: a partial unfolding of the protein; if the motif region is binding to the intracellular membranes, it should be released from the membranes for recognition; or disassemble from the protein-protein interaction [21]. Posttranslational modifications in proteins missing change the incomplete motif into a perfect KFERQ motif. Such as, by phosphorylating the tyrosine (Y), cysteine (C) or serine (S) residue can contribute a missing negative charge in incomplete motif, or acetylating the K residue can provide a missing Q [22]. Posttranslational modifications of CMA-targeting motifs provide a method for the regulation of CMA degradation. HSC70 can recognize the substrate proteins in the cytosol through interaction with the KFERQ-like motif [23]. During this process, many co-chaperones are involved in, such as Hsp40, Hsp90 and Hip [24, 25]. The chaperones/substrate protein is targeted to the surface of the lysosomal membrane [26].

## BINDING AND UNFOLDING SUBSTRATE PROTEINS

Once recognized by the chaperone, the HSC70/substrate protein complex is delivered to the lysosomal membrane surface and interacted with the cytosolic tail of LAMP2A [26, 27]. LAMP2A, a single-span lysosomal membrane receptor protein, is a splice variant encoded by the *lamp2* gene [26]. Through its 12-amino acid tail exposes in the cytoplasm, LAMP2A interacts with HSC70/substrate complex [26, 28]. Before translocation, substrate protein needs to be unfolded; this is mediated by HSC70 and its co-chaperones such as Hsp40, Hsp90 and Hip [29].

## TRANSLOCATION INTO LYSOSOME

LAMP2A is located at the lysosome in monomeric form, since substrate proteins only interact with its monomers. This interaction induces the LAMP2A monomers aggregate into a 700 kDa protein complex. During the changing from monomeric to multimeric forms, lys-HSP90 (HSP90 in the lysosome) maintains the LAMP2A stability [24]. The multimeric forms of LAMP2A complexes assist CMA substrate proteins through the lysosome membrane. The substrate proteins translocation through the lysosome also needs the lys-hsc70 (HSC70 in the lysosome) normally resident in lysosomes [30].

## DEGRADATION

After the substrate protein is pulled into the lysosome, it is degraded into amino acid by lysosome hydrolytic enzymes [31]. Lys-hsc70 (HSC70 in the lysosome) induces disassembly of LAMP2A from the multimeric form into the monomeric form, thus the next substrate protein can bind to LAMP2A in a new cycle [24].

## PATHWAY OF CMA

CMA, acts as housekeeping functions, has crucial functions in cellular physiology and pathology. It is involved in cells at a low level under normal conditions. Regulation of CMA means a great deal to the steady state of a cell. However, there is little information on the signaling pathway of CMA. It can be induced by various stressors such as hypoxia [32], oxidative stress [33], DNA damage [34] and prolonged starvation [35]. The calcineurin/nuclear factor of activated T cells (NFAT) signaling pathway was the first CMA-activating signaling pathway identified [36]. Anguiano et al. showed that the activation of CMA depends on a functional retinoic acid receptor alpha (RARα) [37]. Studies have demonstrated that the targeting of rapamycin (mTOR) –protein kinase B (Akt) –pleckstrin homology domain and leucine-rich repeat protein phosphatase (PHLPP) to the surface of lysosomes can directly regulate CMA [38]. The critical mechanisms of CMA respond to mTORC2/PHLPP1/Akt signaling pathway still need further investigation. Many evidences show that there is a tight connection between the CMA and macroautophagy during the degradation of autophagic process. Through upregulating macroautophagy can block the activity of CMA [39]. Likewise, CMA can be induced by blocking macroautophagy [33]. Similarly, CMA pathway is also intimately connected with the ubiquitin–proteasome system [40]. Cross-talk between these pathways has been observed, with one compensating for others if one of them fails. The compensation among proteolytic pathways contributes to maintenance of protein homeostasis.

## CMA SUBSTRATE PROTEIN IN CANCER

**Figure 2:**
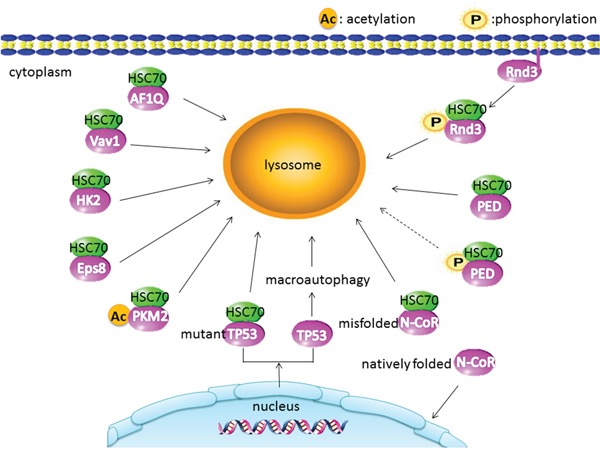
CMA substrate proteins in cancer: the acetylated PKM2 displays a stronger interaction with HSC70 Phosphorylation translocates Rnd3 from membrane to cytosol and promotes Rnd3 interaction with CMA. Unphosphorylated PED binds HSC70 and degradation by CMA. However, phosphorylated PED binds to HSC70 at a low level (dotted line). Misfolded N-CoR is associated with HSC70 and degraded through the CMA. The degradation of mutant TP53 is mediated by the CMA and the degradation of TP53 through macroautophagy.

CMA is an alternative pathway of autophagy mediated substrate protein by HSC70 and LAMP2A; HSC70 recognizes and targets substrate protein bearing a KFERQ-like motif to lysosomal membrane. LAMP2A helps substrates to translocate into lysosome for degradation [18, 29, 30]. These features help us to identify the substrate proteins in the cancer cell. CMA substrate protein plays dual roles in the carcinogenesis and the progress of malignant tumor. It reveals the depth mechanism between the CAM and cancer. Following is the summary of CMA substrate proteins in cancer (Table 1 and Figure 2).

**Table 1:** CMA substrate proteins in cancer

Substrate protein	Role in cancer	Cancer type	Ref.
AF1Q	promoter	ML	[55]
Unphosphorylated PED	suppressor	NSCLC	[78]
Misfolded N-CoR	promoter	NSCLC	[16]
Vav1	promoter	Pancreatic cancer	[101]
PKM2	promoter	NSCLC	[22]
Eps8	promoter	Pancreatic cancer	[134]
Rnd3	suppressor	Gastric cancer	[148]
mutant p53	promoter	Ovarian cancer	[158]
HK2	promoter	Ovarian cancer	[15]

## AF1Q

AF1Q was first identified in acute myelomonocytic leukemia (AMMOL). It is described as a partner of mixed lineage leukemia gene fusion in AMMOL patients [41]. The upregulation expression of AF1Q has been observed in myelodysplastic syndrome (MDS) and acute myeloid leukemia (AML) [42–44]. High expression of AF1Q has been found in hematologic and solid malignancy patients with poor clinical outcomes [43–50]. Overexpression of AF1Q is significantly associated with a higher incidence of distant metastasis [49, 50]. AF1Q not only plays as an oncogenic factor but also has a vital function in apoptosis and chemotherapy drug resistance. Knockdown AF1Q protein in conjunction with the decreased apoptotic cell death is induced by doxorubicin or γ radiation [42, 51]. By upregulation of NF-κB p65, AF1Q can enhance the radiation-induced apoptosis, which may explain the oncogenic mechanism of AF1Q [52–54]. Bioinformatics analysis showed that AF1Q has six amino acids sequence correlated with KFERQ-like motifs. The researches support that AF1Q clearance is involved in CMA pathway, and CMA abnormal may lead to AF1Q related malignant tumors [55]. The molecular mechanisms by which AF1Q influences tumor suppressor gene loss and interacts with oncogene are not fully understood, however, the degradation of AF1Q via CMA pathway offers a promising new treatment option for cancer.

## UNPHOSPHORYLATED PED

Phosphoprotein enriched in diabetes (PED) was first described by Helena over 20 years ago [56]. PED is a 15 kDa molecule consists of a NH2-terminal death effector domain (DED) and a COOH-terminus tail with the extracellular-regulated kinase (ERK) binding site and phosphorylation sites (Ser-104 and Ser-116) [57, 58]. *PED* is a highly conserved gene, which is located on human chromosome 1q21-22, and involves in regulating cellular functions, including survival and metabolism. There are three forms of PED in the cells: unphosphorylated, monophosphorylated and bisphosphorylated PED [59]. Combine unphosphorylated PED with ERK1/2 plays an important role in preventing PED translocation into the cell nucleus, which leads to inhibit the cell proliferation [60, 61]. As an endogenous substrate, PED can be phosphorylated by protein kinase C (PKC) at Ser-104 [62] and protein kinase B (PKB) or Ca^2+^/calmodulin-dependent protein kinase II (CaM kinase II) at Ser-116 [63, 64]. Phosphorylation of PED will result in the inhibition of apoptosis by preventing ERK1/2-binding and enhancing the binding to MORT1 and caspase 8 [62, 63]. Since the role of PED in apoptosis and ERK signal pathway, changes in PED expression may influence oncogenesis, cancer progression and chemotherapeutic sensitivity. PED has now been described as both the tumor suppressor and promoter. Unphosphorylated PED can inhibit proliferation and invasion of cells and correlates with good prognosis [65–68]. PED is known to be upregulated in several cancers and involved in resistance to TRAIL-mediated apoptosis [69–77]. Quintavalle et al. identified PED as a substrate protein of CMA pathway. In cancer cells, CMA targeted degrades phosphorylated PED (tumor promoter) at a reduced extent, however, unphosphorylated PED (tumor suppressor) at normal extent. This phenomenon fills the cancer cell with fuel. In cancer cells, CMA degrades unphosphorylated PED contributes to the resistance of chemotherapy and radiotherapy [78].

## MISFOLDED N-COR

Nuclear receptor co-repressor (N-CoR) was first cloned as the protein associated with unliganded T3R-RXR heterodimers in 1995 [79]. By mediating active repression through nuclear receptors, N-CoR involves in cellular biological processes, including tumor initiation, differentiation and progression [80, 81]. Because of inaccuracy dissociation from nuclear receptors, N-CoR gains the inappropriate function, which causes a variety of diseases, including the human cancer [82]. Numerous studies have mainly focused on the function of N-CoR in transcription regulation [79]. N-CoR has emerged as a regulator of tumor suppressors via transcriptional control [83, 84]. Many evidences support that N-CoR is an essential component of many tumor suppressor proteins [79, 85, 86]. Knockdown of N-CoR suppressed the motility and proliferation of tumor cells [87]. However, the misfolded form of N-CoR loses the tumor suppression role and contributes to the development of non-small cell lung cancer (NSCLC). Bin et al. find misfolded N-CoR is associated with HSC70 and degraded through CMA in NSCLC. Degradation misfolded N-CoR by CMA can suppress the survival and growth in NSCLC cells [16].

## VAV1

VAV family is one of the best-known proteins of Rho/Rac activators [88]. Vav guanine nucleotide exchange factor 1 (Vav1) is a 95 kDa protein of the Vav family (Vav1, Vav2 and Vav3). It is predominantly expressed in haematopoietic cells and consists of several functional domains including CH, DH, PH, SH2, and SH3 domains [89, 90]. Vav1 works as a signal transducer in maturation and immune response [91] and an adapter molecule, promoting interaction between the protein [89, 92]. Vav1 is a key driver of the dynamic regulation of actin cytoskeleton and numerous physical cellular processes of mature hematopoietic cells [93–95]. It is located on chromosome 19p12-p13.2, the domain of karyotypic abnormalities in human solid tumors or hematopoietic malignancies, therefore Vav1 has an essential function in human cancer [94]. Vav1 is specifically expressed in human cancer and plays a major role in carcinogenesis and progression [96–99], it has been defined as an oncogene [100]. Vav1 are regulated by its degradation through an HSC70-chaperone-mediated targeting to the lysosome [101]. Vav1 overexpression increases tumor cell survival, proliferation, and metastasis, thus drugs that targeted degradation Vav1 may be potent inhibitors of tumor cell migration.

## PKM2

Cell proliferation is a process that consumes large amounts of energy, especially in cancer cells. In oncology, cells provide energy at a high rate of glycolysis accompanied with an increasing extrusion of lactic acid in the presence of oxygen, and this is called the Warburg effect [102, 103]. The high aerobic glycolysis has clear metabolic benefits for carcinogenesis and tumor growth. Pyruvate kinase (PK) regulates the last rate-limiting step of the glycolytic pathway and catalyzes the transfer of phosphoenolpyruvate and ADP into pyruvate and ATP [104, 105]. In mammals, the PK family has four known isoforms: PKM1, PKM2, PKL, and PKR [106, 107]. The isoenzyme of PK that allows the upregulation of phosphormetabolite pools in multicellular organisms is PKM2. It is an ancient variant of the pyruvate kinase enzyme found in unicellular organisms such as yeast and *E. coli* [108]. PKM2 possesses the less active dimer and the active tetramer forms [109, 110]. Dimeric PKM2 mainly facilitates the glycolytic intermediates towards biosynthesis and tumor growth, whereas tetrameric PKM2 promotes the activity of glycolysis for ATP production [111]. PKM2 makes an enormous contribution to cancer metabolism. It expresses and actives in cancer cells, which is correlated with the prognosis of tumor [112–114]. Numerous evidences support PKM2 as a tumor marker [115–117]. Through reducing the oxidative metabolism of cells, PKM2 sustains cell growth in hypoxic environments and provides cells with a growth advantage in metabolites [118]. PKM2 is important for cancer cell growth, therefore the inhibitor of PKM2 is very meaningful to the tumor treatment. Shikonin and its analog alkannin can selectively inhibit PKM2 [119]. Peptide aptamers [120] and RNA interference targeting PKM2 [121] also induce a significant decrease in cancer cell proliferation through the inhibition of PKM2. However, PKM2 inhibitors have become disputed since posttranslational modifications of them could promote tumor growth [22, 122]. Lei et al. found the acetylation of PKM2 can enhance the interaction with chaperone. PKM2 acetylation by high glucose reduces the activity of PKM2 and stimulates the degradation of PKM2 via CMA [22]. We can promote the degradation of PKM2 by CMA to reduce the energy of cancer cells by a minimal rate of glycolysis.

## EPS8

The epidermal growth factor receptor pathway substrate 8 (Eps8) was originally characterised as a kinase activity substrate of the epidermal growth factor receptor (EGFR) [123]. Eps8 maps to the human chromosome 12p13.2 and play an important role conserved in evolution [124]. Fazioli et al. demonstrated that Eps8 exists in two isoforms: p97Eps8 and p68Eps8 [123], and most studies referring mainly to the p97Eps8 isoform. Eps8 is universally expressed [124], and overexpression of Eps8 leads to increased mitogenic signaling and malignant transformation [125]. Growing evidence reveals that the expression of Eps8 is elevated in most human solid tumor types and hematologic malignancies, including oral, thyroid, pituitary, esophageal, lung, breast, colorectal, pancreatic, ovarian, cervical cancer and mixed lineage leukemia [126–129]. Furthermore, elevated expression of Eps8 has been variously linked to tumorigenesis, proliferation and migration, and represents a poor prognosis in patients with cancer [126, 130–132]. To improve the prognosis of cancer patients, researchers make great effort to downregulate the expression of Eps8 by chemotherapeutics agents. Yang et al. demonstrated that mithramycin A could suppress tumor cell formation and metastasis in several cancer cell lines through an inhibition of Eps8 [133]. Furthermore, as a new CMA substrate protein, Eps8 has two KFERQ-like motifs for recognizing by HSC70 [134]. Since Eps8 exposes the imperative function in cancer progression, future research may accelerate protein degradation of Eps8 by CMA in cancer cells.

## RND3

Rnd3, also known as RhoE, is a small signaling G protein. It is an atypical member of the Rho GTPase family [135], which involves in diverse cellular functions such as apoptosis, cell polarity and cell-cycle progression [136]. Such functions contribute to cancer cell migration and metastasis [137]. There is some controversy over the function of Rnd3 in tumor biology. Rnd3 has been considered either as an anti-oncogene or an oncogene. Interestingly, Rnd3 differentially expresses in various types of cancer. For example, Rnd3 is overexpressed in pancreatic cancer [138] and NSCLC [139, 140] and under-expressed in prostate cancer [141] and gastric cancers [142]. However, evidences strongly support *Rnd3* may act as an anti-oncogene, such as hepatocellular carcinoma [143], squamous cell carcinoma [144], breast cancer [145, 146], prostate cancer [141], colorectal carcinoma [147], and lung cancer [145]. Researchers demonstrated that Rnd3 is a novel substrate protein for CMA, and the degradation of Rnd3 by CMA pathway can maintain cell proliferation in gastric cancer [148]. Thus, finding the natural products to reduce the degradation of Rnd3 through CMA as a treatment for cancer may evolve over the next several years.

## MUTANT TP53

Tumor protein p53 (TP53), also known as p53, was first identified in 1979 as an SV40-binding protein by Lionel Crawford [149]. The *TP53* maps to the human chromosome 17p13.1 and encodes a 53 kDa phosphoprotein [150]. TP53, the ‘guardian of the genome’, acts as a checkpoint control for cell cycle, cell differentiation, programmed cell apoptosis or death, and DNA synthesis and repair [151, 152]. The TP53 protein serves as a major barrier against cancer development, and the inactivation of TP53 pathway is found in human tumors [153, 154]. The majority of these mutations in *TP53* are single-base substitution and loss of alleles [155]. TP53 mutations result in loss of tumor suppressor activities and gain of oncogenic functions [156]. TP53 mutants are contributing to tumor survival, proliferation, genomic instability, disruption of tissue architecture, angiogenesis, invasion, migration, and metastasis [157]. The researchers found that degradation of mutant TP53 is specifically mediated by the CMA pathway [158]. Thus, reducing the level of mutant TP53 proteins via CMA represents an attractive anticancer strategy.

## HK2

Hexokinases (HKs) catalyze the cardinal process in glycolysis [159]. There are four isoforms of HK in mammals: HK1, HK2, HK3, and HK4 [160, 161]. HK2 is associated with the mitochondrial membrane, so loss HK2 can inhibit glucose metabolism and destroy the mitochondria [162]. Among these isozymes, the high expression of HK2 has been observed in lung, breast, pancreatic, ovarian cancers and hepatocellular carcinoma, and this is usually associated with poor prognosis [163–167]. HK2 is regulated by the transcription factors such as p53, Myc, and HIF-1 [168]. Besides, the effective anticancer drug 3-bromopyruvate (3BP) is a structural analog of pyruvic acid, which plays the inhibitory effects on HK2 [169–171]. HK2 is characterized as an oncoprotein since its role in tumor onset [164]. The researchers found HK2 degrades via CMA [15]. This suggests CMA may manipulate cellular metabolism and it may be a means of anticancer therapeutics.

## SUBSTRATE GENES TARGET NETWORKS SUGGEST CMA INVOLVES IN WARBURG EFFECT OF CANCER

**Figure 3:**
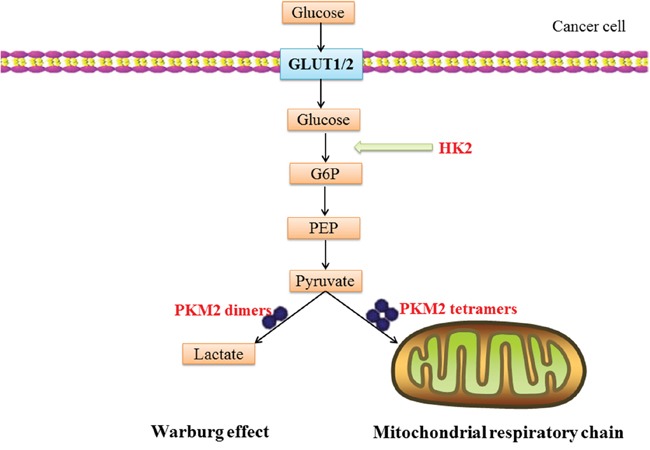
A schematic illustration of CMA substrate proteins involve in Warburg effect: glucose translocation through the plasma-membrane by glucose transporters (GLUT1/2) is rapidly phosphorylated to glucose-6-phosphate (G6P) by HK2 PKM2 dimers and tetramers possess low and high levels of pyruvate kinase activity, respectively. PKM2 dimer redirects the conversion of pyruvate to lactate; the PKM2 tetramer promotes the oxidative phosphorylation through the mitochondria respiratory chain. The Warburg effect describes the enhanced conversion of glucose to lactate by tumor cells, even in the presence of adequate oxygen that would ordinarily be used for oxidative phosphorylation.

To identify the relevance of the cancer-associated substrate proteins, we analyze the substrate proteins by the Database for Annotation, Visualization and Integrated Discovery (DAVID). DAVID is a publicly available tool designed by the Laboratory of Immunopathogenesis and Bioinformatics, which is able to introduce the gene on KEGG pathway [172]. It can get access at http://david.abcc.ncifcrf.gov. The cancer-associated substrates were inputted into the Functional Annotation tool of DAVID. Table 2 lists the KEGG pathways association with substrate genes. Among the five pathways, the molecular pathway entitled “hsa05230: central carbon metabolism in cancer” shows a great association with substrates in cancer. The schematic illustration of CMA substrate proteins involve in Warburg effect is illustrated in Figure 3.

**Table 2:** List of five KEGG pathways and relative genes

KEGG pathway	Genes	P-Value	Benjamini
hsa05230:Central carbon metabolism in cancer	HK2, PKM, TP53	5.0E-4	3.1E-2
hsa04930:Type II diabetes mellitus	HK2, PKM	2.8E-2	5.8E-1
hsa00010:Glycolysis/Gluconeogenesis	HK2, PKM	3.8E-2	5.5E-1
hsa01200:Carbon metabolism	HK2, PKM	6.4E-2	6.4E-1
hsa04919:Thyroid hormone signaling pathway	N-CoR, TP53	6.4E-2	5.6E-1
hsa05202:Transcriptional misregulation in cancer	N-CoR, TP53	9.4E-2	6.4E-1

In this pathway, HK2 and PKM2 are involved in Warburg effect by catalyzing glycolysis. In the beginning of the 20th century, Otto Warburg noticed that cancer cells show a high level of glycolysis accompanied with lactic acid fermentation even in the presence of oxygen [173]. This process is known as the Warburg effect [109]. This indicates that cancer cells prefer aerobic glycolysis for energy, rather than mitochondrial oxidative phosphorylation. The Warburg effect contributes to the survival and proliferation of tumor cell [174]. Previous studies reveal a critical role of PKM2 in tumorigenesis by promoting the Warburg effect. In cancer cells, knockdown of PKM2 increased oxygen consumption, reduced glucose uptake and lactate production [175]. HK2, a CMA substrate, catalyzes the first crucial step of glucose metabolism by phosphorylation of glucose to glucose-6-phosphate (G6P) [176]. The KFERQ-like motif of HK2 also binds to glucose molecules. The CMA motif of HK2 is hid in the protein when there is a glucose molecule. In other conditions, this motif is exposed for the recognizing by HSC70 [177]. CMA influences the glycolysis of malignant cell via degrading the enzymes of glycolytic and TCA cycle. Thus the maintenance of the Warburg effect requires functional CMA in cancer cells [14]. CMA substrate proteins may develop into a new anticancer therapeutic approach through decreasing the glycolysis of the cancer cell.

## CONCLUSIONS AND FUTURE PERSPECTIVES

CMA is one of the autophagy-lysosome pathway which targets substrate proteins to the lysosomal membrane one by one for their degradation [7, 19]. CMA substrate protein plays a crucial role in cancer. However, substrate proteins found in CMA pathway may also degrade through other proteolytic systems, such as proteasomes or macroautophagy. For example, AF1Q and Rnd3 are degraded through the CMA and the proteasome-based system [55, 148]. This phenomenon suggests that CMA and proteasome system can co-regulate the substrate proteins. Prolonged glucose starvation can induce the degradation of mutant TP53 via macroautophagy [178]. Likewise, the cross-talk is existing in CMA and macroautophagy [179]. Mutant TP53 maybe select the mode of degradation between macroautophagy and CMA under different conditions. Different states of substrate proteins also influence the degradation through CMA, such as posttranslational modifications and different mutant alleles. In fact, acetylation contributes to the degradation of PKM2 by CMA [22].

By assessing the role of CMA substrate protein in cancer, we find PKM2 and HK2 are the key enzymes in glycolysis which contribute to the Warburg effect of the malignant cell. Abundant data indicate that blockade of CMA decrease the levels of glycolytic enzymes [180]. This result is somewhat counterintuitive because we insist that inhibiting CMA would lead to an accumulation of CMA-dependent glycolytic enzyme. Since blockade of CMA pathway is still debated, targeting to the CMA substrate proteins provides a new direction of cancer therapy. The fact that the CMA substrate protein motif is not in strict conformance with a five-amino acid residue sequence [20, 181], this makes it possible to create a motif out of an imperfect motif acquiring more effective recognition through posttranslational modifications [78, 182, 183]. Such as, acetylation the K residue can provide a missing Q, which explains that acetylation can increase the targeting of some glycolytic enzymes interaction with HSC70 [22]. HSC70 targets unphosphorylated PED, changing phosphorylated PED into unphosphorylated PED may suppress tumorigenesis. Selective modulation of cancer-associated CMA substrate proteins can also aid study the molecular mechanism of tumorigenesis. Such as, PKM2 is a crucial downstream protein of mammalian target of rapamycin (mTOR) [184]. Disruption of mTOR may suppress oncogenic PKM2-mediated tumorigenesis. Research shows a cross-talk between macroautophagy and CMA [185], mTOR also plays a crucial role in regulating macroautophagy [186]. PKM2 may as a key protein between macroautophagy and CMA for further cancer-associated study.

In principle, approximately 30% of proteins in the cytoplasm contain the HSC70-targeting sequence [187, 188], more CMA substrate proteins are pending further verification. CMA substrate proteins assist us to comprehend the relationship between CMA and cancer cells. Selective modulation of cancer-associated CMA proteins by posttranslational modifications shows the potential for cancer therapy.
